# A Threshold Homomorphic Blockchain Architecture for Secure and Scalable IoT Sensor Data Aggregation

**DOI:** 10.3390/s26216904

**Published:** 2026-10-31

**Authors:** Narendra Kumar Dewangan, Mounira Msahli

**Affiliations:** LTCI Lab, Department of Computer Sciences and Networks (INFRES), Telecom Paris, Institut Polytechnique de Paris, 19, Place Marguerite Perey, CS 20031, F-91123 Palaiseau Cedex, France; narendra.dewangan89@gmail.com

**Keywords:** IoT, Paillier encryption, threshold decryption, Hyperledger Fabric, authenticated aggregation, reproducible evaluation

## Abstract

We present Φ-PHE-BC, an authenticated weighted Paillier aggregation architecture with threshold recovery and a Hyperledger Fabric 2.5.16 implementation. Signatures bind integrity-sensitive sensor fields and ledger-backed nonces prevent replay. The single-host testbed uses two peer organizations and one Raft orderer, with three-of-five recovery in local off-chain shared objects. Twenty fresh-key functional runs recovered the expected aggregate and rejected 200 malformed-input evaluations. A separate 20-run campaign measured preparation of four readings through verified recovery at 4566.48±18.33 ms, excluding dealer setup and configuration commitment. A randomized matched comparison used 20 trials per mode and offered rate, with 16 records per transaction. At 50, 100, and 150 offered transactions/s, encrypted processing achieved 49.37±0.14, 95.36±5.55, and 93.09±11.57 valid transactions/s including drain; authenticated plaintext achieved 49.97±0.01, 99.89±0.03, and 149.36±1.18, respectively. These mean and sample-SD results quantify processing and representation overhead under shared-host conditions; plaintext does not provide equivalent confidentiality. Separate load and block-cut studies document backlog and generator drops. Graph connectivity, threshold recovery and Raft ordering retain distinct assumptions. Distributed-holder fault recovery, peer-count scaling, and a confidentiality-equivalent comparator remain unevaluated.

## 1. Introduction

IoT services exchange sensitive readings among devices, operators, and data consumers. A permissioned ledger can record auditable, tamper-evident processing decisions, whereas encryption can protect specified data contents under explicit key and adversary assumptions. Auditability does not imply confidentiality of transaction metadata, and confidentiality does not by itself establish lawful use or truthful sensor measurements. The relevant engineering problem is therefore the joint treatment of authenticated inputs, authorized aggregate recovery, communication, and processing cost in a permissioned setting; Bitcoin proof of work is not the baseline for the implementation studied here.

Paillier encryption supports addition and public integer-weighted aggregation over ciphertexts [1,2]. Related work applies homomorphic processing to healthcare IoT, federated learning, and transport data [3,4,5,6,7,8,9]. However, each application makes its own choices about key custody, recovery authorization, computation validity, and network conditions. These choices must be compared explicitly rather than inferred from the presence of a blockchain.

The contribution is a precisely scoped architecture, a reproducibility-oriented implementation, and a single-host Fabric evaluation, not a new encryption primitive or a new Byzantine consensus protocol. The revised analysis separates three questions: what encryption and authentication protect; when an overlay remains connected after node removal; and which processing costs an implementation measure. These questions are related but are not interchangeable security guarantees.

The contributions are as follows:

1. A complete authenticated transaction specification with public weighted aggregation and dealer-based threshold recovery using established cryptographic techniques.
2. A separation of graph path resilience, quorum intersection, protocol progress assumptions, and serial service-time modeling. The graph result is a standard conditional connectivity statement, not a new liveness equivalence.
3. An explicit local benchmark protocol and public transcript verifier linking configuration, signed inputs, aggregation, recovery, workload accounting, and run-level summaries and matched plaintext processing overhead.
4. A conditional incentive model that makes the equal-cost assumption visible and distinguishes mathematical preference from deployed enforcement.

We assess these contributions against the closest work. We make no priority claim for simply combining Paillier, signatures, threshold recovery, and graph analysis.

Table 1 positions the design against relevant lines of work. A missing dimension below means it is not established by the comparison presented here, not that a cited paper lacks all formal security analysis.

## 2. Literature Review

The closest antecedent for distributed recovery is threshold-Paillier access control [2]; the encryption primitive itself is established [1]. Application studies in healthcare, cloud-edge sharing, transport, and carbon accounting motivate different data and trust boundaries [3,8,9,12,13]. Surveys and cross-chain studies help distinguish data confidentiality from system-level processing and interoperability [14,15]. These studies should be compared by who sees a reading, who holds recovery authority, and what an output reveals, rather than by treating all HE–ledger combinations as one equivalent baseline.

A second distinction concerns supported computation. Paillier permits additive aggregation and multiplication by a public integer, whereas FHE or approximate HE supports different operation sets under different parameter and correctness constraints [10,16,17]. Work on publicly verifiable HE, compiler verification, homomorphic signatures, and implementation safety addresses distinct verification layers [18,19,20,21,22]. A signature on a ciphertext authenticates submitted bytes; it is not a proof that the encrypted reading is in range or that the sensor is truthful.

A third distinction separates protocol functionality from experimental equivalence. Reputation, auditing, masking, and privacy-preserving learning can alter the trust and communication model [4,5,6,23,24,25]. Selective encryption and searchable data access solve additional application problems [26,27]. Collaborative analytics and other PHE applications broaden the design space but do not automatically translate into throughput gains for this workload [28,29,30]. Consequently, comparing different processors, consensus, key sizes, workloads, and security settings yields no cross-system speed advantage.

### Why Paillier, and When an Alternative Is Preferable

Public-key encryption allows clients to submit independently encrypted readings, while an aggregator uses public integer weights without receiving decryption shares. Threshold recovery distributes the authority to reveal a result. These are functional reasons for the choice; modular exponentiation and ciphertext expansion remain substantial costs. Additive masking and secret-sharing designs can be less expensive on constrained devices. Their setup, communication, dropout recovery, and collusion assumptions depend on the specific protocol and cannot be dismissed categorically. The present package does not benchmark those alternatives and therefore does not establish Paillier’s efficiency superiority.

The gap pursued here is a traceable relationship among input authentication, recovery authority, graph assumptions, and measured processing scope. We analyze graph connectivity separately from consensus progress, whose assumptions depend on the chosen protocol. We discuss future quantum migration briefly in Section 8.6, rather than treating it as a current contribution.

## 3. System Model

Table 2 defines the notation used below. The device communication graph $G_{d}$ and validator overlay $G_{v}$ are distinct. The current implementation represents devices as synthetic clients of a central queue, not as a physical or emulated wireless device network.

**Definition** **1** (Validator overlay)**.** *Let $G_{v}=\left(V_{v},E_{v}\right)$ be a finite simple undirected graph with $N=|V_{v}|\geq 2$. Its vertices are logical validators. Vertex connectivity $\kappa (G_{v})$ concerns path survival after vertex removal, and $\mathrm{diam}(G_{v})$ is the unweighted hop diameter when the graph is connected. Delay parameters used by the local simulator are configured costs, not measured physical link delays.*

(1) $$f_{\max}=\left\lfloor \frac{N-1}{3}\right\rfloor ,\;Q=\left\lfloor \frac{2N}{3}\right\rfloor +1.$$

Equation (1) specifies a fault-budget convention and a strict two-thirds voting threshold. It does not supply a consensus implementation or a proof of its safety and progress.

The four generated families in the corrected package refer to $G_{v}$ only:

- Tree: vertices 0,…,N−1 with edge (⌊(i−1)/2⌋,i) for each i>0; a balanced binary heap tree.
- Star: center 0 adjacent to every other vertex.
- Mesh: the complete graph $K_{N}$, not a grid or torus. Its diameter is one for N>1.
- Scale-free model: an undirected Barabási–Albert graph with attachment count two, reduced to N−1 only for very small test graphs; the generation seed is recorded.

We save all edges, diameter, and vertex connectivity for each run. We do not assume a deterministic logarithmic-diameter guarantee for every scale-free realization. Trees and stars have connectivity one; their short paths do not tolerate the arbitrary removal of a critical validator. A separately augmented overlay would be a different graph and must be recorded as such. Figure 1 depicts these definitions, not measured network performance.

**Definition** **2** (Capability classes)**.** *The classes $D_{I},D_{\mathrm{II}},D_{\mathrm{III}}$ identify configured capabilities, not hardware performance established by MHz/RAM thresholds. Direct mode $D_{\mathrm{II}}$ and $D_{\mathrm{III}}$ clients must support the configured encryption and signature operations. A $D_{I}$ client unable to authenticate its own transaction requires a separately specified authenticated gateway path.*

No unauthenticated device mode is accepted. In a gateway design, the gateway may see the reading and signs under its own registered identity; this is gateway attestation, not a device-origin signature. The corrected package rejects direct $D_{I}$ execution and marks the gateway transport as unimplemented. No physical constrained-device timing is inferred from host measurements or artificial slowdown factors.

The intended cryptographic adversary is probabilistic polynomial-time and may observe public ciphertexts, signed records, and authorized aggregate outputs. Device keys must be uncompromised for the corresponding authenticity claim. Dealer setup and public-key registration are trusted. A coalition with at least *t* decryption shares can recover arbitrary ciphertexts under that key; aggregate-only recovery is an authorization policy, not a restriction imposed on such a coalition by Paillier.

The local fault model is narrower than arbitrary Byzantine consensus: selected validators withhold participation, and reliable authenticated routing is idealized within reachable honest components. The implementation has no equivocation, locking, view-change, or adversarial-network protocol. An adversary allowed to delay every message forever cannot simultaneously satisfy an eventual-delivery assumption. Graph statements and conditional protocol discussion in Section 6 keep these assumptions separate. All private shares coexist in one process, so this experiment does not establish operational isolation against host compromise.

The target ledger evaluation is a local Hyperledger Fabric 2.5.x network with Raft. Raft provides crash-fault-tolerant ordering, not Byzantine-fault-tolerant ordering [31]. Byzantine key-share withholding and malformed-share rejection are application-level questions; they do not upgrade Raft’s fault model. The measured deployment uses Fabric 2.5.16, two endorsing peers in separate organizations and one Raft orderer; details are recorded in Section 8. One orderer provides no orderer-crash tolerance.

## 4. Framework Design

### 4.1. Framework Parameterization

Parameterization identifies what the implementation can execute before selecting any protection layer. In particular, cryptographic key size, the number of recovery shares, voting quorum, block configuration, and optional mechanism flags have different meanings. The framework rejects unsupported choices instead of silently selecting a weaker or simulated mechanism.

**Definition** **3** (Implemented instance)**.** *The implemented baseline instance is*

(2) $$\begin{array}{cc} F & =(G_{v},D,\Phi ,\Lambda ,\Pi ),\\ \Lambda & =(n,\mathrm{ECDSA}\;\;:\;P256,H,\eta ,\nu ),\\ \Pi & =(N,t,Q,B,B_{\mathrm{bytes}},T_{b},T_{\mathrm{age}},T_{\mathrm{future}}). \end{array}$$

*Here,* Φ *is an integer-weighted sum modulo n; H is SHA-256; and $T_{\mathrm{age}}$ and $T_{\mathrm{future}}$ are freshness-policy limits. BFV/CKKS parameters and a noise-flooding statistical target are not baseline parameters because these mechanisms are not implemented. The key-size policy is enforced at setup; the selection algorithm does not receive an unused security-parameter input.*

### 4.2. Cryptographic Primitives

For n=pq and g=1+n, Paillier encryption and public weighted aggregation are

(3) $$c_{i}={\left(1+n\right)}^{v_{i}}r_{i}^{n}\;\mathrm{mod}\;n^{2},\;r_{i}\overset{\$}{\leftarrow }Z_{n}^{*},\;c_{A}=\prod_{i\in I}c_{i}^{w_{i}}\;\mathrm{mod}\;n^{2}.$$

The decrypted result is $\sum_{i\in I}w_{i}v_{i}\;\mathrm{mod}\;n$. An ordinary integer sum requires a justified bound preventing wraparound; ciphertext well-formedness alone cannot prove that private values meet that bound. Reading values in the diagnostic workload are synthetic integers from one smart-meter cohort.

ECDSA P-256/SHA-256 authenticates canonical messages. The trusted registry pins the device public key, permitted weight, and cohort. The transmitted record contains neither a plaintext reading nor encryption randomness. Encryption confidentiality under DCR and signature unforgeability are inherited assumptions, not new primitives introduced by this work.

The corrected construction uses the established safe-prime dealer-based threshold scheme [32]. It does not use Shamir interpolation over an unrelated fixed 521-bit field, and the public combiner does not receive a full private key. Research equality-of-exponent transcripts are verified for partial recovery; these transcripts are distinct from optional plaintext-range or computation-validity proofs.

### 4.3. Cryptographic Layer Selection

Algorithm 1 is a capability check for the implemented baseline, not a proof that every requested function can be supported by an automatic composition of primitives. Authentication remains mandatory for every accepted path. Optional ZKP is never inferred solely from membership in $D_{\mathrm{III}}$.
**Algorithm 1** LayerSelect**Input:** capability *D*, function Φ, gateway flag *g*, optional proof flag *z*.
**Output:** supported authenticated layer set, or an explicit unsupported status.If Φ is not a public integer-weighted sum, return UNSUPPORTED_FUNCTION.If *z* is true, return UNSUPPORTED_PLAINTEXT_ZKP.If $D=D_{I}$, require *g* and return GATEWAY_DESIGN_NOT_IMPLEMENTED; direct execution is rejected.If $D\notin \{D_{\mathrm{II}},D_{\mathrm{III}}\}$, reject the capability.Return {Paillier, mandatory ECDSA authentication, verified threshold recovery}.

No completeness proposition is asserted for unsupported gateways, bridge functions, or plaintext proofs. Threshold-share verification is mandatory within the recovery procedure and is not controlled by the optional plaintext-proof flag.

### 4.4. Protocol Phases

#### 4.4.1. Phase 0: Trusted Threshold Setup

A dealer chooses distinct safe primes $p=2p^{\prime }+1$, $q=2q^{\prime }+1$, sets n=pq, $m=p^{\prime }q^{\prime }$, and Δ=N!. It selects *d* and a sharing polynomial such that

(4) $$\begin{array}{c} d\equiv 0\;(\mathrm{mod}\;m),\;d\equiv 1\;(\mathrm{mod}\;n),\\ s_{i}=f\left(i\right)\;\mathrm{mod}\;nm,\;\deg f=t-1,\;f\left(0\right)=d. \end{array}$$

The public parameters include a square verification base and per-validator verification keys. Private shares are assigned to logical holders. This is trusted-dealer setup, not distributed key generation. Python does not guarantee erasure of dealer secrets, and the benchmark is one process.

#### 4.4.2. Phase 1: Identity and Weight Registration

An operator-controlled registry pins each sensor ECDSA public key, weight, and cohort. In Fabric, the Org1 administrator registers an immutable key epoch in chaincode; the sensor registry is distinct from Fabric MSP enrollment. Fabric CA 1.5.17 containers support the test-network identity setup, but sensor certificate issuance and revocation are not evaluated.

#### 4.4.3. Threshold Recovery Specification

For an aggregate $c_{A}$, holder *i* generates the partial and a ciphertext-bound equality-of-exponent transcript. The public combiner validates the transcript, excludes invalid shares, rejects duplicate indices, and requires at least *t* distinct verified shares. For a selected set *S* of size *t*,

(5) $$\begin{array}{cccc} c_{i}^{\prime } & =c_{A}^{2\Delta s_{i}}\;\mathrm{mod}\;n^{2}, & {𝓁}_{i} & =\Delta \prod_{j\in S\setminus \{i\}}\frac{-j}{i-j}\in Z, \end{array}$$

(6) $$\begin{array}{cccc} u & =\prod_{i\in S}{\left(c_{i}^{\prime }\right)}^{2{𝓁}_{i}}\;\mathrm{mod}\;n^{2}, & M & =\frac{u-1}{n}{\left(4\Delta^{2}\right)}^{-1}\;\mathrm{mod}\;n. \end{array}$$

For the recovery-share check, the witness is $x_{i}=\Delta s_{i}$, the bases are *v* and $c_{A}^{4}$, and the targets are $v^{x_{i}}$ and ${\left(c_{i}^{\prime }\right)}^{2}$. The SHA-256 challenge binds the key fingerprint, ciphertext, validator index, partial, and commitments. If $b_{x}=\mathrm{bitlength}\left(\Delta n^{2}\right)$, proof randomness has $b_{x}+256+128$ bits and the response is an unreduced integer; these are computational proof parameters, not flooding-mask parameters. No new information-theoretic guarantee is inferred from the 128-bit slack.

The code checks the domain of the Paillier recovery function. It neither reconstructs nor falls back to a full decryption key at the combiner. Threshold protocol correctness follows the established construction and its setup assumptions; functional tests alone do not prove confidentiality.

#### 4.4.4. Secure-Parameter Recovery Evidence

Twenty actual 2048-bit, three-of-five functional runs generate verified partials and recover the expected aggregate. Partial generation takes 1210.44±16.58 ms and verification/combination 862.66±18.77 ms (mean ± sample SD). Insufficient-share rejection is checked locally. These measurements do not change the holder population or prove isolation, malicious-holder tolerance, or recovery from operational withholding.

#### 4.4.5. Phases 2–4: Sensing, Signing and Validation

The client encrypts a reading and signs the following unambiguous, typed message:

(7) $$M_{i}=\mathrm{Encode}\left(\mathrm{domain},ID_{i},c_{i},w_{i},ts_{i},{\mathrm{nonce}}_{i},\eta_{i},\nu \right),\;\sigma_{i}=\mathrm{ECDSA}\;\;:\;P256.{\mathrm{Sign}}_{sk_{i}}\left(M_{i}\right).$$

ECDSA applies SHA-256 internally. Encoding is compact sorted ASCII JSON: identity, weight, and timestamp are integers; the ciphertext is a decimal string; timestamps use integer milliseconds; the nonce is 128-bit lowercase hexadecimal. The cohort and key epoch are also bound. No unkeyed hash is treated as a signature.

Algorithm 2 describes acceptance. Signing $w_{i}$ prevents in-transit modification but does not authorize a dishonest client to choose an arbitrary weight. Signing $ts_{i}$ prevents modification but does not replace a freshness-policy check.
**Algorithm 2** Transaction verification**Input:** signed record; trusted key/weight/cohort registry; current time; accepted-nonce state.Parse the canonical types; require $0<c_{i}<n^{2}$ and $\gcd(c_{i},n)=1$.Resolve the registered public key; require the correct key epoch and authorized weight/cohort.Reconstruct exactly $M_{i}$ in Equation (7) and verify $\sigma_{i}$; reject on failure.Require $t_{\mathrm{now}}-60,000\leq ts_{i}\leq t_{\mathrm{now}}+2000$, with all times in milliseconds.Atomically reject an already accepted pair $\left(ID_{i},{\mathrm{nonce}}_{i}\right)$; update the corresponding acceptance/commit state according to the local path.Admit the record. The quorum simulator also rejects duplicates within a batch and previously committed pairs before voting.

The standalone verifier’s replay state lasts for its process lifetime; simulator validators retain committed pairs within one run. We do not implement persistent ledger replay prevention across restarts. Invalid records do not acquire accepted-nonce state merely by presenting a previously unseen nonce.

#### 4.4.6. Phases 5–6: Aggregation and Local Commitment

The batcher selects one explicit set *I*, constrained by transaction count, encoded bytes, and timeout. All signature/policy checks and the weighted product in Equation (3) refer to this same set. Logical validators are executed serially. Withheld vertices are excluded; under ideal authenticated routing the largest surviving connected component supplies votes. A strict quorum is required. Successful commitment updates a local hash chain and nonce state. This procedure is a synchronous research simulation, not PBFT, Tendermint, or Fabric ordering.

#### 4.4.7. Phase 7: Threshold Recovery

After commitment, the required holders generate and verify the partials specified in Equations (5) and (6). The public combiner requires *t* distinct verified shares and records whether aggregate recovery succeeded.

#### 4.4.8. Phases 8–9: Verification Scope and Retrieval

The baseline does not execute an optional plaintext-range or aggregate-computation proof. Public transcripts permit independent checks of signatures, weighted ciphertext arithmetic, and threshold combination under trusted public parameters. Aggregate availability is recorded separately from commitment. A failed quorum and an unrecovered committed aggregate are distinct outcomes; neither is silently counted as a successful recovered transaction.

The deployment uses enrolled application identities and MSP policies, endorsing peers running deterministic chaincode, a Raft ordering service, and committing peers. Sensors retain the signed payload defined above; Fabric transaction-envelope authentication is an additional layer, not a substitute for authenticating the sensor’s ciphertext, weight, timestamp, and nonce. The JavaScript chaincode reproduces canonical verification and policy checks, binds each submission to its key epoch, and uses ledger-backed replay protection. Fabric transaction timestamps must be checked under an explicit policy; nondeterministic peer wall-clock reads must not determine endorsement outcomes. Secret Paillier shares remain outside the ledger and chaincode; a recovery service combines only verified partials for an identified committed aggregate. The functional execution verifies this path. Timestamp consistency is checked against the client-supplied proposal timestamp; it is not an independently trusted wall clock. The client additionally checks freshness against its wall clock.

An application batch of sensor records is not necessarily a Fabric block. Experiments must report whether one sensor submission or one aggregate batch constitutes a Fabric transaction, and report sensor records/s separately from valid committed Fabric transactions/s. The application batch limit and Fabric channel block-cut parameters must be varied and recorded separately.

### 4.5. Optional Paillier–BFV Bridge

The bridge is an unevaluated design direction. No encrypted hash of the Paillier secret key is asserted to be a usable proxy re-encryption key. A decrypt-and-re-encrypt service would require explicit authorization for the input, a specified source-key provisioning or threshold-recovery path, authenticated destination-key binding, plaintext processing inside the trusted boundary, output encryption, and key revocation/rotation procedures.

TEE integrity and its attestation mechanism would be additional security dependencies. Attestation can identify a measured enclave configuration; it is not, by itself, an independent proof of arbitrary computation correctness. Such a claim would require a separate proof relation, transcript and verifier. A concrete threat model must cover or explicitly exclude side channels, rollback, and host-assisted leakage. Provisioning a full source key to an enclave would also change the trust-boundary threshold; it cannot be treated as the same operational guarantee.

Accordingly, we do not retain any bridge confidentiality proposition or end-to-end composition proof for an unspecified construction. The corrected executable rejects bridge requests. Optional BFV/CKKS computation, re-encryption, attestation, and proof performance are not included in baseline timing or evidence.

### 4.6. System Architecture

Figure 2 shows the implemented local boundary. Public parameters reach clients and holders; private shares reach holders only; the trusted key registry supplies identity and policy checks. Key management is connected to its recipients. The deployed chaincode is JavaScript, not Go.

Figure 3 starts with threshold setup before client registration and submission, then separates commitment from recovery and records failure paths. We keep this reference diagram separate from the actual Fabric binding in Figure 4.

Figure 2 and Figure 3 describe only the executed local reference path. The intended Fabric binding adds MSP-backed client identity, endorsement/chaincode execution, Raft ordering and peer commit validation, as specified in Section 6 and the deployment requirements above. The actual deployment is shown separately in Figure 4.

Figure 4 describes the actual Fabric roles separately from the reference simulator.

## 5. Security Analysis

Paillier’s encryption confidentiality is inherited under the DCR assumption for an appropriate modulus and a classical polynomial-time adversary [1]. Public homomorphic manipulation does not turn IND-CPA encryption into a chosen-ciphertext-secure or data-validity mechanism. The observed aggregate output is an intentional disclosure: ciphertext privacy must not be phrased as hiding a value already revealed by an authorized aggregate or auxiliary information.

Threshold recovery uses an established construction [32]. Any security claim must include the concrete scheme’s trusted-dealer, corruption-threshold, setup, and proof-model assumptions. This research implementation has not received an independent cryptographic audit, and a single host holding every logical share provides no operational isolation from that host.

The earlier noise-flooding theorem and dependent end-to-end hybrid bound are withdrawn. A postulated statistical-distance condition on noisy exponents did not specify how masks were generated, correlated, cancelled and hidden under collusion. Deterministic post-processing of that condition alone did not prove a correct and private joint protocol. No flooding width, mask capacity, or target $\varepsilon_{\mathrm{nf}}$ is reported as an implemented parameter. Partial-proof nonce randomness is not a flooding mask on a decryption share. No information-theoretic share-privacy guarantee is claimed for the evaluated baseline.

**Proposition** **1** (Authentication of complete transaction contents)**.** *Assume an uncompromised registered signing key, an unambiguous encoding, and EUF-CMA security of the configured signature mechanism. Acceptance of a new message in Equation (7) not signed by that device implies a signature forgery. Modifying identity, ciphertext, weight, timestamp, nonce, cohort, or key epoch changes the authenticated message.*

**Proof.**  The verifier reconstructs exactly the encoded message used by the signer. A changed field produces a distinct encoded message because field types and boundaries are fixed. Acceptance of a signature on that new message contradicts the stated unforgeability assumption. This is an inherited signature argument, not a claim that an unkeyed digest authenticates a sender.    □

Freshness, replay rejection, registration, and weight authorization are separate acceptance predicates. An authenticated client can still submit an unauthorized weight or false reading; a compromised key is outside the corresponding authenticity premise. The local process-lifetime nonce state must not be described as persistent ledger replay protection. The implemented negative tests exercise these distinctions, but do not prove cryptographic hardness.

## 6. Network Resilience and Performance Analysis

**Theorem** **1** (Connectivity under vertex removal)**.** *Let $G_{v}$ be a finite connected simple graph on N≥2 vertices and 0≤f≤N−2. Then, removal of every set of at most f vertices leaves a connected graph if and only if*

(8) $$\kappa (G_{v})\geq f+1.$$

**Proof.**  If the minimum vertex cut has size at least f+1, no allowed removal is a disconnecting cut. Conversely, a cut of size at most *f* is itself an allowed removal leaving nonempty disconnected components. This is the standard definition-based graph result.    □

This graph result alone does not establish Byzantine message authenticity, reliable routing, admission fairness, quorum intersection, locking, leader replacement, eventual delivery, or transaction inclusion. A disconnected overlay can still contain a quorum-sized component; therefore, a partition argument alone does not establish a universal necessary condition for all notions of consensus progress.

For a specified consensus protocol, graph conditions must be combined with its own safety/progress theorem and routing/synchrony assumptions. For the strict quorum in Equation (1), two quorums intersect in at least two 2Q−N vertices. That numerical intersection does not enforce honest voting or locking behavior. The local simulator idealizes routing, permits only withholding and makes no general Byzantine consensus claim.

Let $N_{o}$ denote the number of orderers in the channel’s Raft consensus set. Its majority count is $\lfloor N_{o}/2\rfloor +1$; the corresponding crash-count budget is $\left\lfloor \left(N_{o}-1\right)/2\right\rfloor$, subject to the protocol’s communication and eventual-progress assumptions [31]. This is not the simulator’s strict two-thirds voting rule. The threshold (t,N) applies only to Paillier shareholders, while the Fabric endorsement policy governs endorsement. Orderers, endorsing peers, and shareholders need not coincide. The abstract tree/star/mesh/scale-free graphs are not claimed to be measured Fabric network topologies.

### 6.1. Latency and Throughput Model

Under an explicitly serial scheduling assumption, a modeled aggregate-availability service time can be written as

(9) $$S\left(G_{v}\right)=r_{\mathrm{comm}}\mathrm{diam}\left(G_{v}\right)\tau_{\mathrm{hop}}+S_{\mathrm{auth}}+S_{\mathrm{agg}}+S_{\mathrm{share}}+S_{\mathrm{combine}}+S_{\mathrm{other}}.$$

The local code uses three abstract communication sleeps; these are prescribed model costs, not measured packets. Authentication, aggregation, partial generation, and combination are executed and timed. Queue wait and batch formation add delay before service. Client encryption/signing and scheduled-to-submission lag are recorded separately. Submission-to-commit excludes subsequent recovery, whereas submission-to-availability includes it.

For a full serial batch of *B* transactions and a stable service time *S*, B/S is a service-capacity estimate under that model, not an identity for observed throughput. Measured committed throughput is

(10) $$\hat{X}=\frac{\#\{\text{measured-cohort}\;\mathrm{valid}\;\mathrm{transactions}\;\mathrm{committed}\;\mathrm{in}\;\mathrm{the}\;\mathrm{observation}\;\mathrm{window}\}}{T_{\mathrm{obs}}}.$$

Pipelined Fabric processing would additionally involve endorsement, ordering/batching, validation and ledger/database updates, client communication, and queueing. The subsequent Fabric measurements time their observable composites; they do not infer individual internal-stage costs from the serial simulator.

Let $k=\lceil \log_{2}n\rceil$ and M(2k) denote the arithmetic cost of multiplication modulo $n^{2}$. Aggregation performs a number of modular products proportional to the batch size, plus public-weight exponentiation work. With bounded non-negative weights, a rough bit-operation accounting is $O(B\left(1+\log\left(1+w_{\max}\right)\right)M\left(2k\right))$, rather than the earlier unsupported $O(n_{d}\log n)$ wall-time claim. Recovery cost depends on *t*, proof arithmetic, and serial/parallel scheduling.

For the actual graph generators, the binary tree has logarithmic height, the star diameter is two for N>2, and the complete mesh diameter is one. Scale-free diameter is computed per realization. These communication observations are not universal end-to-end throughput bounds or BFT guarantees.

The client must record proposal/endorsement, submission, and peer-confirmed commit status using the actual available API [33]. Ordering acknowledgement alone does not establish a valid ledger update. Throughput must count valid committed transactions within a stated observation window; invalid commits, timeouts, and unresolved submissions must be recorded separately. End-to-end availability additionally includes the defined aggregation and threshold-recovery stages. Stage times not exposed by the platform must be reported as unavailable or measured composites, rather than inferred as separate network or orderer timings.

#### Observed Timing Boundaries

The original 20-run functional campaign measured four-record proposal-to-valid-commit latency at 2045.12±5.13 ms. A separate 20-run fresh-key campaign subsequently measured preparation of four readings through verified recovery as one monotonic interval, 4566.48±18.33 ms. Its proposal-to-valid-commit component was 2079.90±5.85 ms. The campaigns are not pooled. Section 8.3 specifies the complete interval and exclusions. No synchronized internal ordering, validation, database, or network-packet timestamps were collected; client-observed composites do not isolate those internal costs.

### 6.2. Communication Accounting

A hypothetical all-to-all voting protocol with $r_{v}$ voting phases would have logical message volume on the order of

(11) $$C_{\mathrm{logical}}=\sum_{i\in I}|T_{i}|+r_{v}N\left(N-1\right)|V|+t|D|+|H_{B}|.$$

Here, |D| includes the actual partial-decryption transcript, not a nonexistent flooded-share wrapper. Physical byte-hops would also depend on routing paths, retransmissions, transport framing, and fanout. The present single-process simulator does not send those voting messages, and its sleeps do not measure communication volume. Equation (11) is conditional protocol accounting, not a measured cost or an implemented PBFT guarantee.

## 7. Conditional Incentive Analysis

A binary incentive model is useful only within its stated payoff assumptions. Let $R_{i}$ be the honest reward, $G_{i}$ the deviation gain, $p_{i}$ the probability a penalized deviation is detected, and $P_{i}$ the penalty. Honest and deviating computational costs may differ:

(12) $$U_{i}\left(H\right)=R_{i}-\alpha C_{i}^{H},\;U_{i}\left(D\right)=G_{i}-\alpha C_{i}^{D}-p_{i}P_{i}.$$

The condition for strict preference for *H* is

(13) $$p_{i}P_{i}>G_{i}-R_{i}+\alpha \left(C_{i}^{H}-C_{i}^{D}\right).$$

If payoffs satisfy this inequality for every profile of other players, it gives strict dominance; if parameters are merely fixed expectations, it gives only the corresponding conditional preference. Under equal costs and certain detection, the common cost cancels. Deviations that avoid cryptographic work do not satisfy that equal-cost premise. Equality gives indifference, not strict dominance.

The local package implements no detector, reward mechanism, penalty enforcement, or empirical equilibrium study. A degree-dependent reward is an additional policy choice, not a consequence of graph connectivity. This analysis is therefore a conditional design observation, not evidence that the implementation induces an all-honest equilibrium or remains incentive-compatible under arbitrary cryptographic overhead.

## 8. Implementation and Verification Evidence

Two evidence layers are reported separately: earlier local reference diagnostics with simulated validator voting/delays, and the subsequent actual Fabric deployment. Fabric results count peer-confirmed valid commits; they do not measure a distributed BFT protocol. The synthetic readings are generated inputs, not live sensor acquisition or recorded-dataset replay.

The ledger evaluation executes Hyperledger Fabric 2.5.16 on one Apple M5 host. Peer/orderer binaries and Docker report ARM64; no cross-architecture emulation is reported. All containers and the load client share physical resources. Host RAM is 24 GiB, whereas the captured Docker VM exposes 10 CPUs and 8,319,504,384 bytes of memory (approximately 7.75 GiB). Multiple containers do not constitute independent physical deployment sites.

Table 3 summarizes the actual deployment. Logical reference diagnostics use a separate process and are not Fabric measurements.

Each chaincode container has a 2 GiB memory limit. Peers and the orderer have no explicit container memory limit; no CPU quota or pinning is configured. All services share the Docker VM. The bridge subnet is 172.18.0.0/16 with gateway 172.18.0.1. Image identifiers are retained in the evidence. Historical utilization and thermal monitoring are unavailable for the original campaigns; the subsequent isolated two-peer campaign retains separate Docker resource samples.

### 8.1. Executed Functional Checks

The regression suite reports 33 passing tests. Coverage includes changes to every signed field, signature forgery, unauthorized signed weights, stale/future timestamps, replay races, duplicate records, ciphertext-domain checks, insufficient/duplicate/malformed partials, strict quorum at counts divisible by three, and star-center removal. At test size, every one of the ten three-of-five share subsets recovers each tested message. These are functional checks, not a computational security proof.

The package additionally saves public parameters, registered public keys, signed ciphertext records, and partial-recovery transcripts. The independent verifier checks signatures, registered policies, batch arithmetic, distinct-share recovery, and duplicate commitment under trusted inputs. All 12 workload smoke runs and the single 2048-bit diagnostic were checked. Plaintext readings, encryption randomness and private shares are not written to those public records.

#### Actual Fabric Validation

Twenty corrected fresh-key runs each submitted four authenticated records, verified a valid commit (code 0), checked the public ciphertext product, and recovered the expected weighted sum. All 200 negative-input evaluations returned their expected chaincode errors, and 20 replay checks passed the strict client check. These are endorsement-stage rejections via evaluate, not invalid transactions committed in blocks. One representative valid aggregate has transaction ID ae7f9c54555c4fd481b20325724ede8f63801573538dbaabe7a50eede84501f2 in block 17. Earlier attempts used a faulty Buffer-valued error response and counted transport timeouts as rejections; they are excluded from security-success counts. Chaincode 1.1 returns a string error, and the corrected client requires the expected rejection reason. The corrected repeated-run archive retains actual errors and commit references.

Unsupported legacy values (3120 transactions/s, 410 ms end-to-end latency and derived cross-system gains) remain withdrawn. The matched comparison in Section 8.4 executes both modes on the same network and reports their natural payload and processing differences. Because the plaintext mode lacks confidentiality, this experiment does not establish superiority over an equivalent privacy-preserving scheme.

### 8.2. Topology and Workload Diagnostics

The generators in Section 3 produce the four graphs in Figure 1. These are logical validator overlays, not device topologies or packet-network experiments. Their displayed connectivity values are graph computations for the depicted realizations.

Earlier simulator checks are retained as software diagnostics in the supporting package: 12 short workload runs using intentionally insecure 128-bit test keys, one 2048-bit diagnostic, and four small-key microbenchmark runs. They do not establish secure-parameter throughput or deployment scalability. Their detailed plots are omitted from the main evaluation to keep the measured Fabric evidence central. The 33-test regression suite and saved public transcripts support implementation checks under trusted setup, not production-security certification.

### 8.3. Complete Processing Interval

Twenty sequential fresh-key trials measure preparation of four readings through encryption, signing, valid commitment, committed read-back, independent ciphertext-product verification, and verified three-of-five recovery. The weighted sum is 1020 in every trial. All 20 commits are valid; 200 negative-input evaluations, 20 replay checks, and 20 insufficient-share checks return their expected outcomes. The earlier pilot is excluded.

Table 4 reports a single Python monotonic interval and selected nested components. It includes file I/O, Node.js startup, and Gateway connection, but excludes dealer key generation, device setup, configuration commitment, and negative tests. Components are already contained in the complete interval and must not be added to it again. The original functional campaign remains separate.

Dealer setup, outside this interval, averages 132.48±98.31 s. Shares remain local Python objects; the results do not include distributed-holder communication. Internal ordering, validation and database stages are not independently instrumented.

### 8.4. Matched Plaintext and Encrypted Comparison

The comparison executes authenticated plaintext and authenticated Paillier aggregation on the same persistent Fabric network, with identical registry, signing identity, two-organization AND endorsement, 16-record batches, configured MaxMessageCount 20, and 2 s timeout. Each mode/rate has 20 trials with a 30 s offer window. The 60 matched pairs are randomly ordered; mode order within pairs is also randomized, and a two-second pause separates settings. The client cap is 4096 outstanding requests. One valid warmup transaction per mode is excluded. Public pool values match between modes; encrypted mode reuses a pool of 64 ciphertexts with fresh signatures, timestamps, and nonces.

Table 5 and Figure 5 report run-level means and sample SD. Across 360,000 scheduled requests, 357,805 started transactions have unique IDs, valid commit status and matching aggregate results, with no invalid or unknown commits, endorsement errors, or aggregate mismatches. All 2195 cap drops occur in encrypted mode at 150 offered transactions/s. Plaintext contributes 180,000 valid transactions; encrypted mode contributes 177,805. Full transaction events were checked against per-run counts, means, nearest-rank p95, and throughput denominators.

Plaintext lacks confidentiality. Natural payload lengths and cryptographic execution both differ, so this is a combined processing/representation comparison, not isolated modular-arithmetic cost or a closest-work privacy-preserving benchmark. Encryption, signing preparation, and recovery are outside proposal-to-commit load timing. At low load, block formation and payload cutting can affect latency; the lower encrypted mean at 50 transactions/s does not establish lower cryptographic cost. At high load, queueing and cap drops qualify throughput. A shared host, one key epoch, and a growing ledger preclude independent-deployment inference.

#### Separate Isolated Two-Peer Campaign

A subsequent two-peer laboratory campaign completed 120 settings, recording 359,153 unique valid transactions without invalid or unknown commits, endorsement errors, or aggregate mismatches. Its 847 generator drops at 150 transactions/s in encrypted mode comprise 842 cap drops and five missed schedules. A preceding interrupted attempt is excluded and preserved separately. Docker monitoring records 588 resource samples for the completed campaign. This is an additional two-peer execution, not a comparison across peer counts; its results are not pooled with the original network. Four- and six-peer experiments are not included.

### 8.5. Executed Fabric Workload Studies

The executed campaigns use a 2048-bit modulus and actual Fabric Gateway commits. Application batch size, block-cut settings, and arrival rate are separate variables; results below retain the realized sample counts.

Fabric channel block cutting is controlled separately through its message-count/byte limits and timeout. The application batch size must not be called the Fabric block size. If one Fabric transaction carries multiple sensor records, report both units. Record warmup, steady observation and drain periods, and distinguish a committed Fabric transaction from successful decrypted-aggregate availability.

#### 8.5.1. Workload Construction and Instrumentation

Each Fabric transaction contains an application batch of signed ciphertext records. The load client uses a pre-encrypted pool of 64 valid Paillier ciphertexts, fresh timestamps/nonces, and freshly computed signatures. Reusing ciphertexts is disclosed: encryption is outside the load timing and this is not an evaluation of fresh per-reading encryption throughput. One P-256 key is registered under the synthetic device IDs; the test does not emulate heterogeneous independent devices. All weights are one in workload campaigns, while the functional cohort separately verifies nonuniform weights. Every successful transaction result is checked against an independently computed public ciphertext product.

The scheduler targets fixed interarrival times, records per-request scheduling lag, signs records before proposal creation, and permits concurrent Gateway submissions. It saves a transaction ID before endorsement and records submission and terminal status separately. Latency starts at proposal construction and ends after commit-status/result processing, excluding signing preparation. MaxInflight is 256 in pilots and 4096 in boundary/block-cut studies. A cap drop is a scheduled request not started, not a ledger rejection. Backlog denotes client-observed outstanding requests, not direct observation of an internal orderer queue. Throughput including drain is valid completions divided by the actual observation-plus-drain duration; the within-window rate is reported separately. Acknowledgement alone never counts as a valid commit.

#### 8.5.2. Main Repeated Campaign

At MaxMessageCount 10, the completed campaign contains 20 trials per target rate, 30 s of scheduled arrivals each, with 16 records per transaction. All 220,461 started transactions have unique transaction IDs, successful validation codes, and matching ciphertext aggregates. Of 225,000 scheduled arrivals, 4539 are generator-cap drops at target 150 transactions/s. No started transaction has an unresolved commit or validation error in this completed campaign. Table 6 reports run-level means and sample SD, including variability that the short pilots did not reveal.

The mean valid within-offer-window rates are 91.44, 52.70, and 5.90 transactions/s, respectively; the gap from drain-inclusive rates reflects requests completing after the offer interval. Mean within-run p95 latencies are 2.92, 18.48, and 33.50 s. These are averages of per-run quantiles, not pooled quantiles. At 150 transactions/s, outstanding counts reach approximately 4096 at the end of every observation window. Even at 100 transactions/s, several later trials develop large backlogs. Accordingly, the short-pilot conclusion of stable operation at 100 transactions/s does not generalize to all repeated trials.

Figure 6 makes both run variability and outstanding requests visible. These results demonstrate an overloaded region under the tested resource and scheduling conditions, not an exact maximum Fabric capacity. They do not identify whether peer execution, Gateway queueing, host contention or another stage determines the limit.

#### 8.5.3. Preserved Failed Campaign

An earlier 20-repeat attempt stops at its first setting (100 transactions/s): 332 of 3000 scheduled requests start; 2668 are missed schedules; 91 return valid commit status, 75 have unknown commitment; and 166 encounter endorsement errors. Actual elapsed time including delayed completion is 938.64 s. This is a failed execution campaign, retained in the archive and excluded from the completed-campaign means, not silently converted into successful rejections. Its cause is not established; no assumption that those unknown transactions failed on-chain is made.

#### 8.5.4. Application Batching and Configured Block Limits

The initial pilot spans 1, 4, and 16 records/transaction at 1, 5, and 20 transactions/s for 10 s per setting, one trial each. All 780 started transactions commit correctly, with no generator drops. At 20 transactions/s, mean latencies are 257, 252, and 265 ms for the three application batch sizes. These short runs are exploratory and have no independent-run error bars. The higher-load pilot subsequently brackets the overloaded 16-record regime; it is not pooled with the main repeated campaign.

Block-cut campaigns hold the application batch at 16 records and vary the configured MaxMessageCount. Table 7 and Figure 7 report the separately executed limits 5 and 20 at the same target rates and three 30-second repetitions each. PreferredMaxBytes remains 524,288, AbsoluteMaxBytes 103,809,024, and BatchTimeout 2 s. All 26,228 and 26,627 started transactions commit with correct aggregates for limits 5 and 20, respectively. At 150 transactions/s, cap drops total 772 and 373; they are not included in committed-throughput numerators. Actual block occupancy and encoded envelope-size distributions were not separately extracted, so a configured maximum of 20 is not a claim that every block contains 20 transactions. Byte limits can also trigger cutting.

The MaxMessageCount 10 main campaign uses 20 repetitions and a partly different rate grid; it is therefore reported separately. All campaigns execute sequentially on one growing ledger without randomized order or controlled cooling. Descriptive differences are not a statistically controlled causal comparison of block settings. At lighter load, faster block filling can reduce waiting; at overload, queueing dominates. No block-limit superiority claim is inferred.

#### 8.5.5. Unmeasured Scaling Dimensions

No completed comparison across peer counts, orderer counts, endorsement policies, or independent device populations is available. Changing records per transaction changes payload size and synthetic device IDs together; it is not a controlled device-count scaling study. The earlier graph experiments remain logical reference overlays, not measured Fabric topologies.

#### 8.5.6. Statistical Unit and Campaign Accounting

Error bars and reported SD use run-level observations. The main campaign comprises 20 sequential 30-second trials per rate on one persistent network, one key epoch and one pre-encrypted pool; they are not independent network restarts. Within-run p95 uses the nearest-rank quantile of valid transaction latencies; averages of those quantiles are not pooled p95 values. No confidence intervals implying independent deployments are reported. The original campaigns use fixed rate order and lack controlled cooldown/thermal measurements; the later matched campaign randomizes pair and mode order and includes a two-second pause between settings. Neither provides independent deployment replications. Warmup is one excluded valid transaction per campaign; each setting drains all started requests to terminal client status. Unknown commits are not counted as valid.

No deliberate Raft orderer, peer, or channel disruption is assumed in this initial workload study. Any crash-recovery experiment requires a separately controlled and documented test. A key-share withholding experiment is not a Byzantine-consensus test, and changing the holder threshold does not change Raft’s ordering guarantees.

### 8.6. Limitations and Future Work

The evidence includes an actual single-host Fabric deployment and separate local reference diagnostics, but no physical IoT deployment. Reference validator operations and recovery shares reside in one Python process; only the reference simulator uses prescribed communication sleeps. Fabric peers/orderers run as real containers. Clients use host cryptography rather than heterogeneous constrained hardware. Dynamic membership, mobility, intermittent wireless links, malicious-dealer behavior, equivocation, and consensus recovery remain outside the evaluated scope. Generalized scalability and BFT guarantees are not inferred from these diagnostics.

A DI gateway and sensor certificate service require separate development and validation. The actual ledger binding implements persistent replay state, while application timestamp checks remain conditional on client-supplied proposal time. A TEE bridge would introduce enclave/key-management and verification assumptions; optional plaintext proofs require a concrete relation and implementation. Neither is part of the current baseline timing. Noise flooding is not included, and its unsupported theorem has been withdrawn.

Post-quantum migration is future work. Replacing authentication or transport with ML-DSA, SLH-DSA, or ML-KEM [34,35,36] would not make Paillier confidentiality post-quantum secure. A replacement aggregation and threshold construction would require new parameterization, analysis, and measured overhead.

The Fabric workload results establish measured behavior under the stated generator and single-host resource constraints. They do not establish comparative advantage, orderer/peer scaling, independently isolated key holders, or recovery from crashes. The one-orderer deployment cannot survive an orderer crash. Sequential campaigns on a persistent ledger introduce run-order, ledger-growth, resource-contention, and possible thermal confounding; these effects were not experimentally separated. Block limits are configured maxima, not verified occupancy of every produced block.

### 8.7. Code and Data Availability

We supply the corrected implementation source, dependency manifests, benchmark configurations, raw experimental records, and analysis support privately with this revision. These materials retain the distinct functional, timing, workload, and matched-comparison campaigns. The exported evidence excludes enrollment private keys and private threshold shares. Public transcripts do not include private signing keys, threshold shares, or individual sensor plaintexts. Code can be made available after obtaining permission from the project funding authorities.

## 9. Conclusions

This work specifies an authenticated weighted aggregation architecture and a corrected local threshold-Paillier research implementation. The revision separates complete transaction authentication, public aggregate recovery, graph path resilience, and protocol-specific consensus requirements. It withdraws the unsupported information-theoretic noise-flooding and connectivity-to-liveness claims and does not attribute local simulated execution to Hyperledger Fabric.

Functional checks and limited verification traces support execution correctness under the stated setup and registry assumptions. They do not establish comparative speed, production security, or deployment-scale scalability. The matched plaintext experiment quantifies overhead without providing equivalent confidentiality. Closest-work comparisons under matched security assumptions and independently distributed deployments remain prerequisites for stronger performance claims. Future work should address those evidence gaps before extending the design with gateways, stronger computation validity, or post-quantum aggregation.

The actual Fabric 2.5.16 deployment validates signed aggregation and three-of-five local recovery. Complete four-reading preparation-to-recovery timing averages 4.57 s; the matched load study exposes substantial encrypted-processing overhead and queueing at higher offered rates. Repeated load tests show increasing latency and backlog near the tested offered-load boundary; generator limits and temporal variation qualify the results. The measured deployment uses one Raft orderer, so it does not demonstrate Byzantine ordering or orderer-crash recovery.

## Figures and Tables

**Figure 1:**
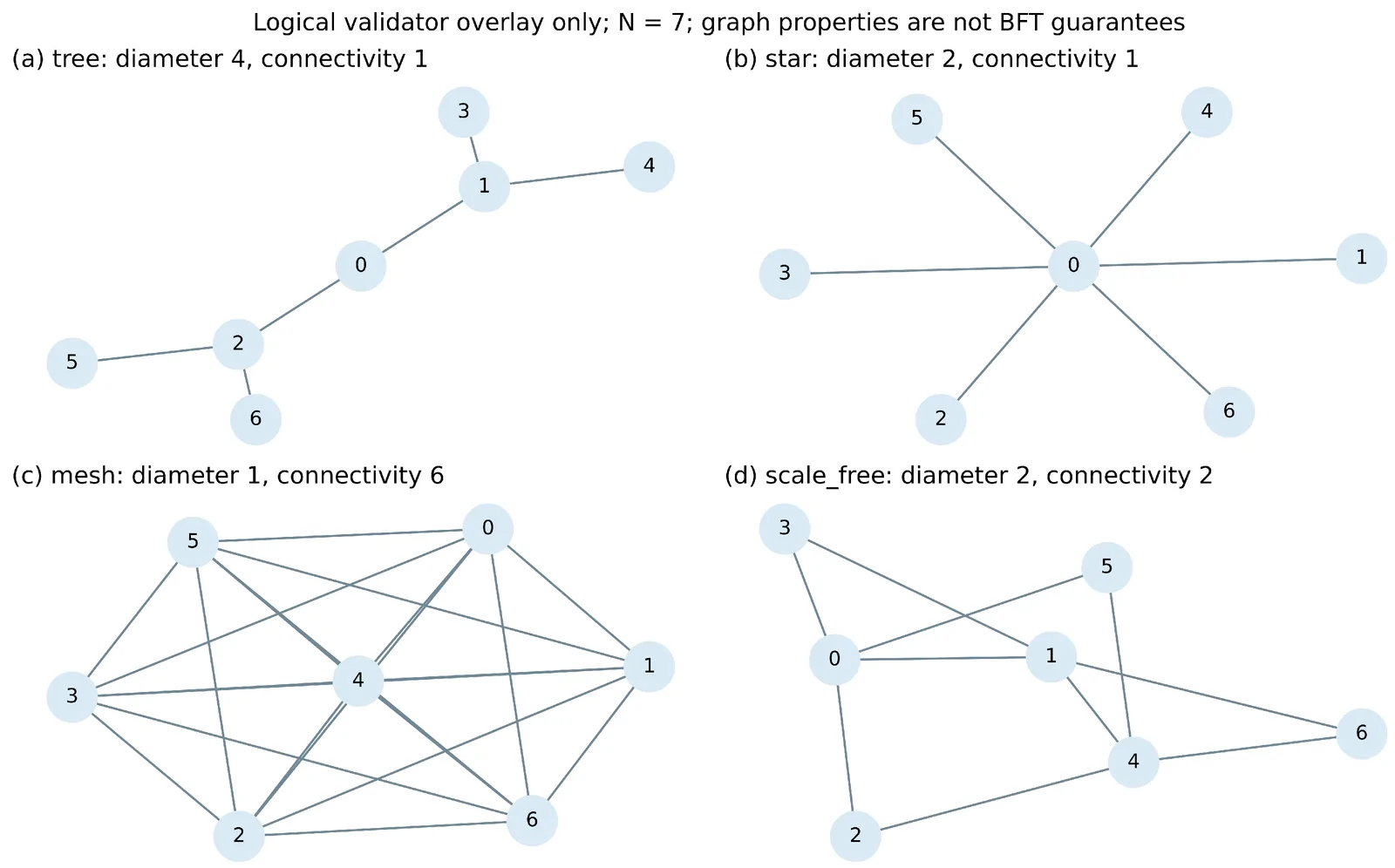
Precisely defined N=7 logical validator graphs: (**a**) balanced binary tree; (**b**) star with center 0; (**c**) complete mesh; and (**d**) Barabási–Albert model with attachment two and seed 42. All subpanels are explicitly labeled. Graph statistics are not BFT liveness guarantees.

**Figure 2:**
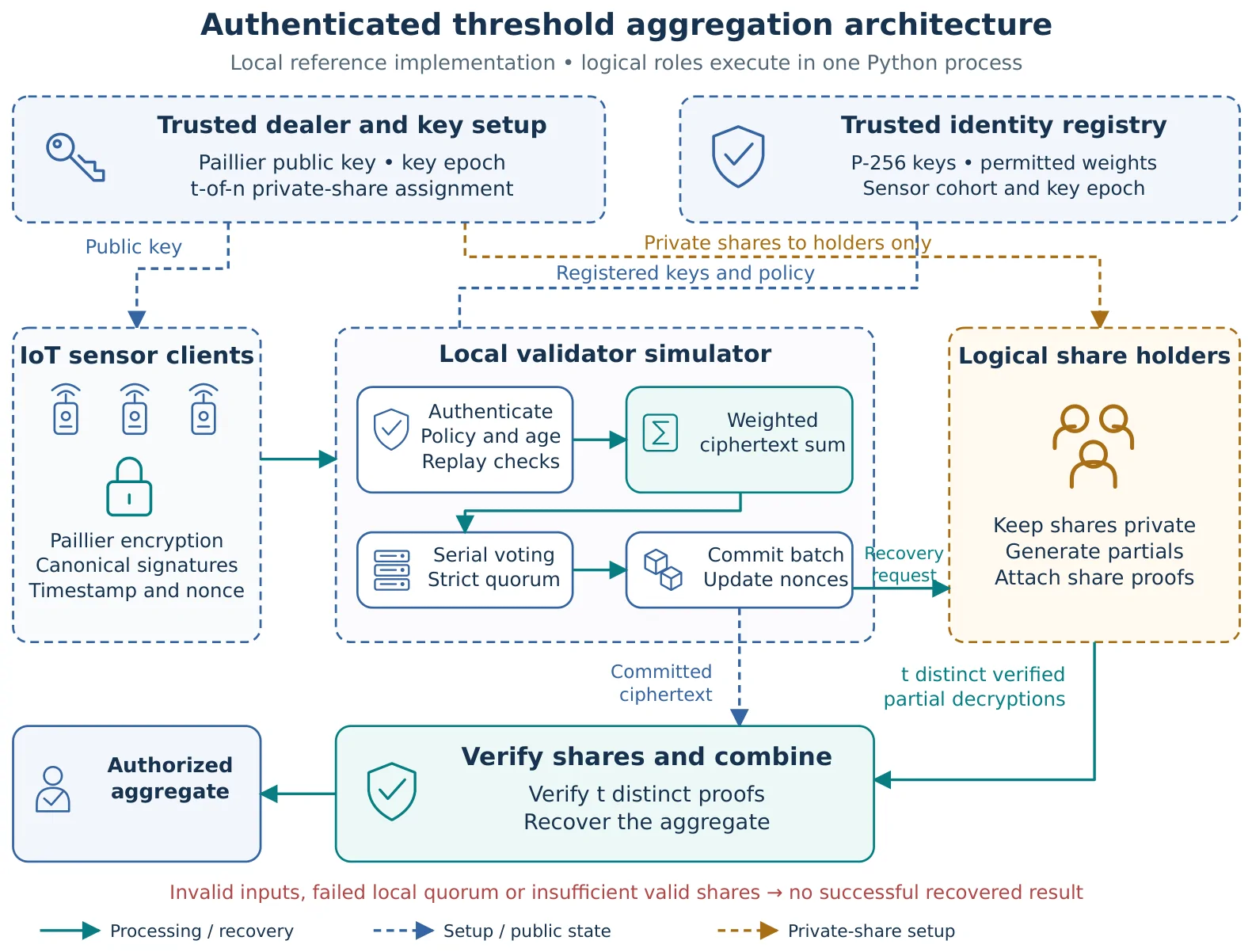
Corrected local architecture. Signatures bind ID, ciphertext, weight, timestamp, nonce, cohort, and key epoch. The local registry is a trusted setup component, not an implemented RA certificate service. At least *t* distinct verified shares are required for public combination; all logical participants are in one process. No Fabric/TEE deployment is represented as evaluated.

**Figure 3:**
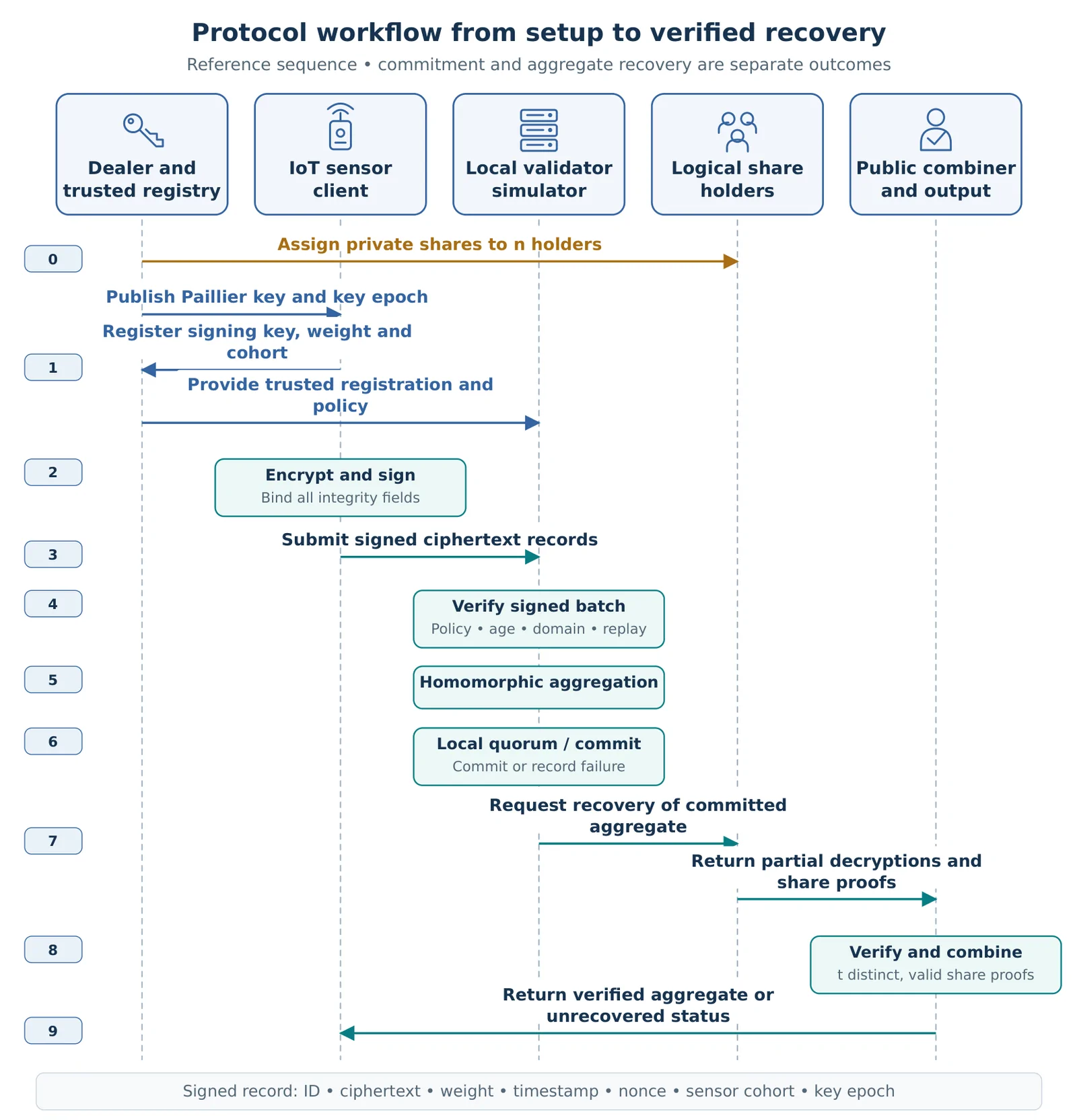
Corrected execution sequence: dealer setup, trusted registration, canonical signing, queueing/batching, checks, local quorum, verified partials, and public combination. Freshness and replay checks do not replace authentication.

**Figure 4:**
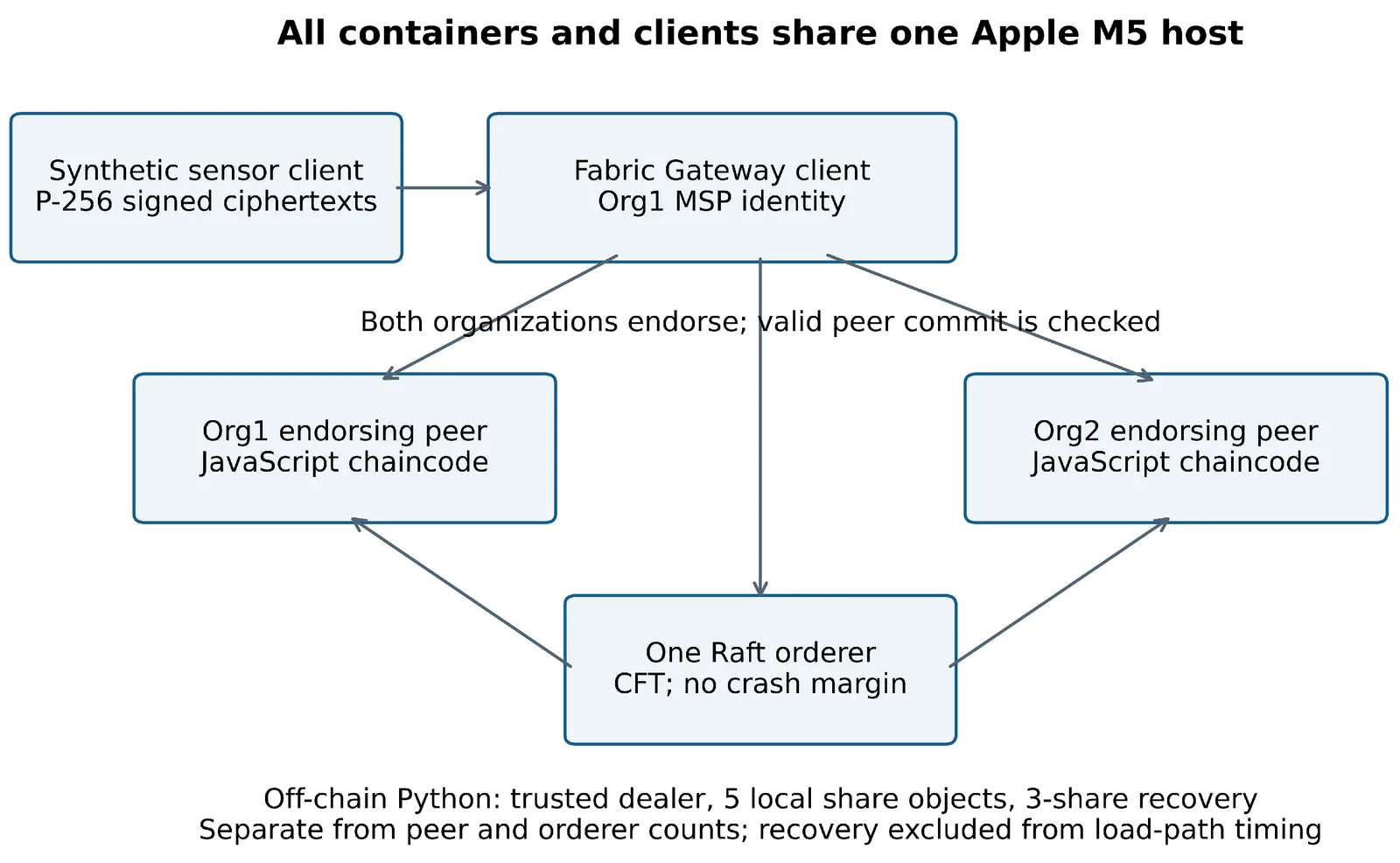
Executed single-host Fabric binding. Two organizations endorse; one Raft orderer orders transactions; peers validate and commit. Threshold holders are local off-chain software objects, not peers or orderers. Sensor signing keys are registered by an MSP-authenticated administrator. Optional TEE/ZKP paths are excluded.

**Figure 5:**
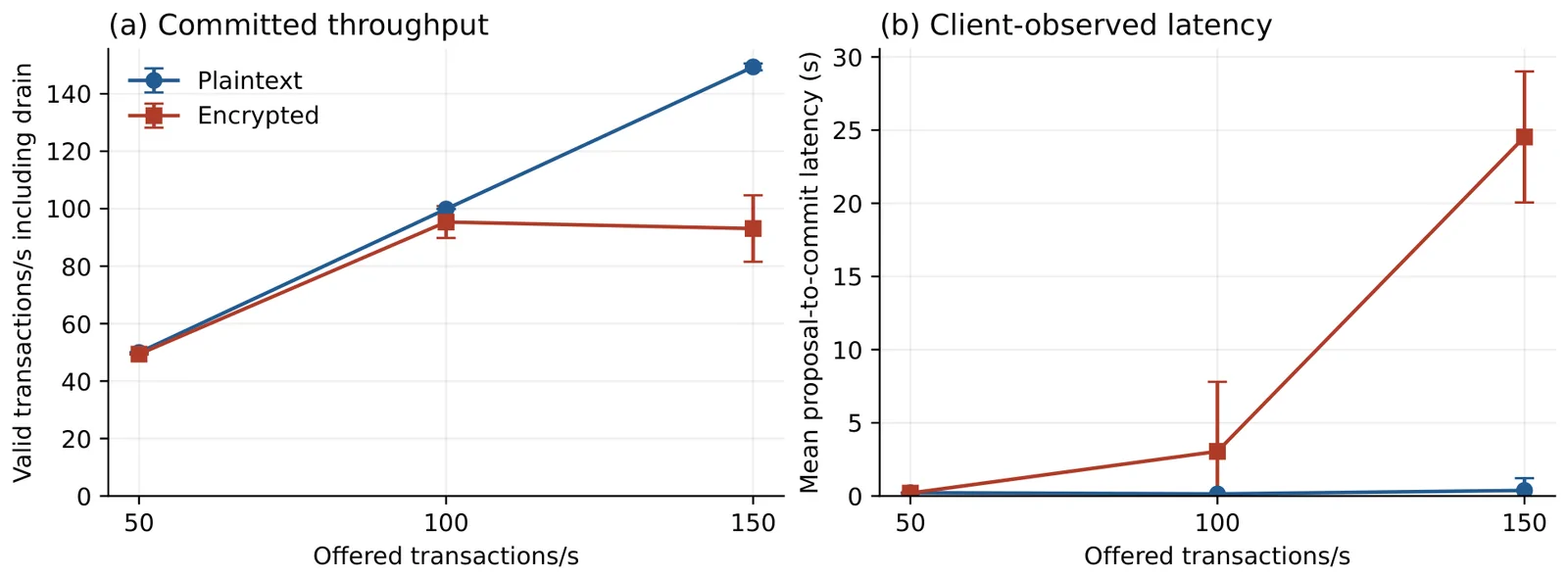
Matched processing overhead: (**a**) valid throughput including drain; (**b**) proposal-to-commit latency. Error bars are sample SD across 20 run-level observations per mode/rate. Lines connect tested points; neither curve is an estimated capacity function.

**Figure 6:**
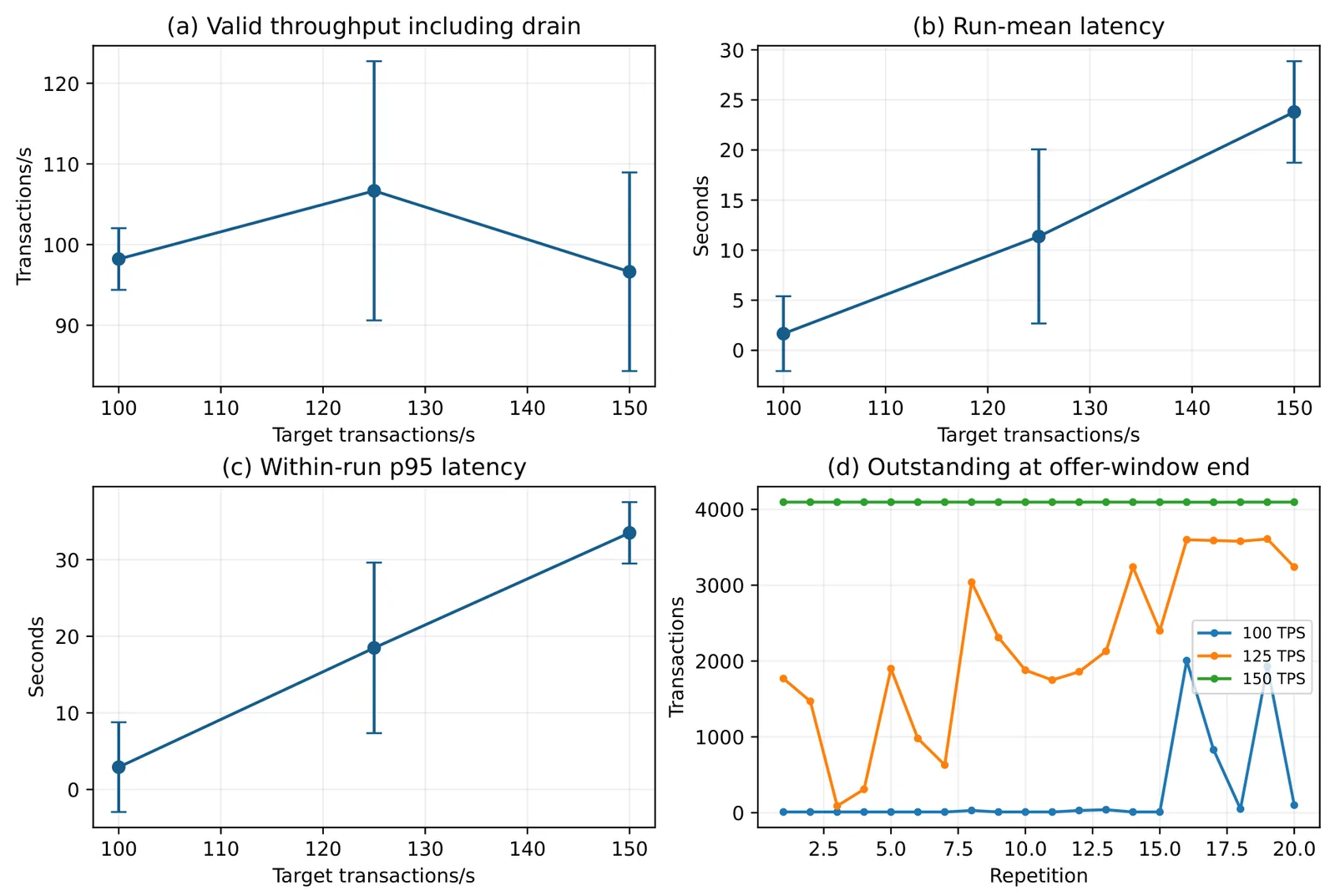
Actual Fabric workload measurements at MaxMessageCount 10 with 16 records/transaction. Panels (**a**–**c**): mean ± sample SD across 20 run-level observations per rate. Panel (**d**): outstanding client requests at the end of each 30 s offer window, before drain. One shared host and persistent network; encryption/recovery excluded.

**Figure 7:**
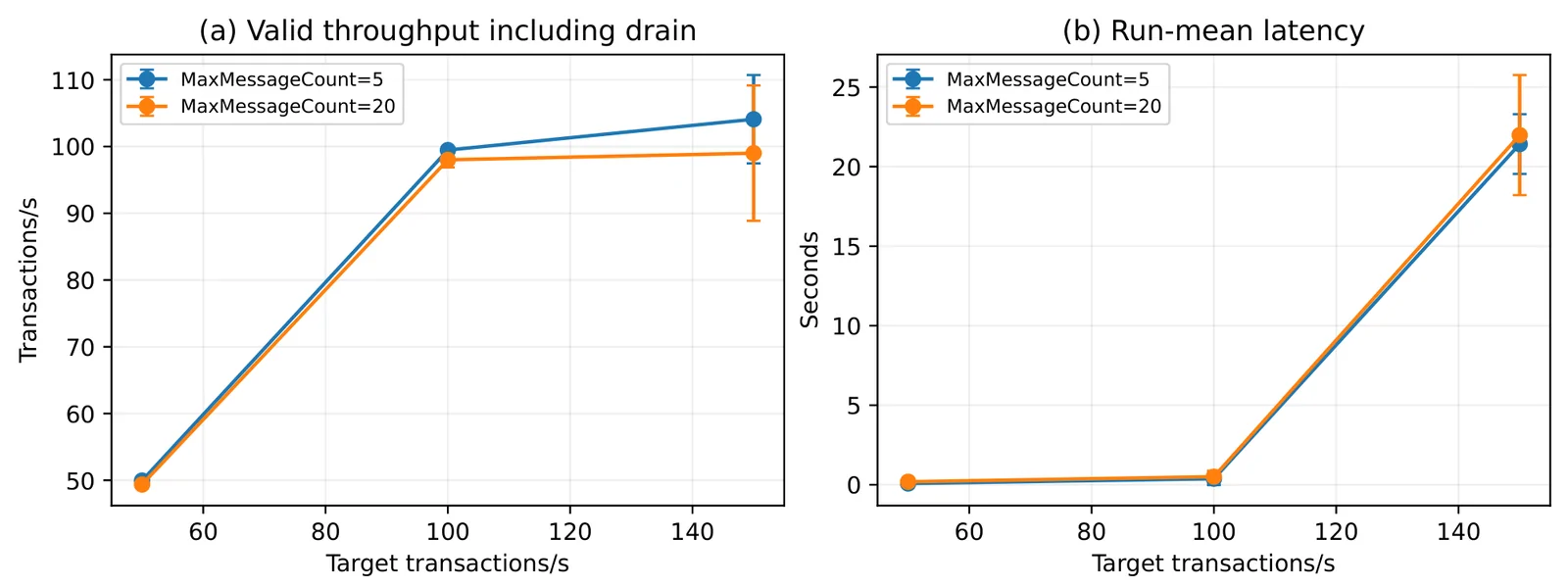
Configured block limits 5 and 20: (**a**) valid throughput including drain, and (**b**) run-mean latency. Three trials/cell, 16 signed records/transaction, 30 s offer interval, and 2 s timeout; error bars are run-level sample SD. Fixed campaign order and generator-cap effects qualify comparisons.

**Table 1:** Closest-work positioning by design question; no numerical speed ranking is inferred.

Related Work	Relevant Overlap	Distinction Requiring Explicit Assessment
Si et al. [2]	Threshold Paillier and ledger access control	Application, authorization, and measured workload differ.
Lakhan et al.; Jiang et al. [8,9]	Protected IoT/vehicular processing with ledgers	Application network assumptions must not be substituted for validator-overlay guarantees.
Wu et al. [10]	FHE-enabled blockchain computation	Supported operations and FHE cost/security parameters differ from additive aggregation.
Latif et al. [11]	Authentication and access control	Authentication does not establish encrypted aggregate-recovery functionality.
This work	Authenticated weighted aggregation and public recovery	Actual Fabric binding and matched plaintext overhead comparison; no distributed scaling claim.

**Table 2:** Notation used in the revised model; the recovery threshold is a count, not a time.

Symbol	Meaning
$G_{d},G_{v}$	Device graph and logical validator overlay, respectively
$N,n_{d}$	Validator count and synthetic device-client count
f,Q	Assumed fault bound and strict voting quorum
n,k	Paillier modulus and its actual bit length
t,Δ	Required recovery-share count and N!
$B,B_{\mathrm{bytes}},T_{b}$	Block count limit, byte limit, and batch timeout
$\tau_{\mathrm{hop}},r_{\mathrm{comm}}$	Configured per-hop delay and abstract communication-phase count
$L_{c},L_{a}$	Submission-to-commit and submission-to-aggregate-availability latency
η,ν	Sensor cohort and cryptographic key epoch/fingerprint
$\sigma_{i}$	Signature on the complete canonical transaction message

**Table 3:** Single-host Fabric configuration for the recorded campaigns.

Setting	Recorded Value
Host/operating system	Apple M5, 24 GiB host RAM; macOS 27.0.1, ARM64
Docker/Compose	Desktop 4.96.0; engine 29.9.1; Compose 5.6.0
Docker VM	10 CPUs; 8,319,504,384 bytes memory; aarch64
Fabric/topology	2.5.16 ARM64; two peer organizations, one peer each; one Raft orderer
Channel/endorsement	phiphe; AND(Org1MSP.peer, Org2MSP.peer)
Chaincode	JavaScript; functional/load studies version 1.1, sequence 2; matched baseline version 1.2, sequence 3
Clients	Node.js 26.0.0; Python 3.14.7; Gateway client API
Encryption/authentication	2048-bit Paillier modulus; ECDSA P-256/SHA-256
Functional recovery	Three of five local Python share objects; off-chain
Block-cut settings	MaxMessageCount 5, 10, 20; PreferredMaxBytes 524,288; AbsoluteMaxBytes 103,809,024; BatchTimeout 2 s
Application batches	1, 4, 16 signed records per Fabric transaction
Database/networking	Embedded LevelDB on both peers; IPv4 bridge fabric_test; IPv6 disabled

**Table 4:** Four-record complete processing interval and nested component measurements. Mean ± sample SD over 20 fresh-key trials; 2048-bit modulus and three-of-five local recovery.

Interval	Time (ms)
Preparation through verified recovery	4566.48±18.33
Batch preparation	281.22±3.54
Gateway process round trip	2199.55±6.28
Proposal to valid commit	2079.90±5.85
Client-observed endorsement	59.57±4.94
Partial-decryption generation	1217.56±8.49
Partial verification and combination	866.46±14.57

**Table 5:** Matched comparison: valid throughput includes drain; latency is the mean of per-run means. Twenty trials per mode/rate, 16 records/transaction. P denotes authenticated plaintext; E denotes encrypted aggregation.

Target tx/s	P Valid tx/s	E Valid tx/s	P Latency (s)	E Latency (s)
50	49.97±0.01	49.37±0.14	0.228±0.005	0.198±0.038
100	99.89±0.03	95.36±5.55	0.143±0.018	3.048±4.752
150	149.36±1.18	93.09±11.57	0.384±0.834	24.532±4.478

**Table 6:** Main Fabric campaign: 16 records/transaction, MaxMessageCount 10, 2 s timeout, 20 sequential trials/rate. Throughput includes drain; latency is the mean of run means.

Target tx/s	Valid tx/s	Latency (s)	Started/Valid	Cap Drops
100	98.21±3.82	1.66±3.73	60,000/60,000	0
125	106.66±16.07	11.37±8.69	75,000/75,000	0
150	96.63±12.31	23.80±5.07	85,461/85,461	4539

**Table 7:** Configured block-limit campaigns: 16 records/transaction, three 30 s trials per cell, fixed 2 s timeout. Means ± sample SD across trials.

MaxMessageCount	Target tx/s	Valid tx/s Including Drain	Mean Latency (s)
5	50	49.96±0.01	0.081±0.003
5	100	99.47±0.45	0.380±0.401
5	150	104.09±6.62	21.42±1.88
20	50	49.37±0.09	0.191±0.006
20	100	98.02±1.14	0.509±0.373
20	150	99.01±10.14	21.98±3.78

## Data Availability

The data presented in this study are available on request from the corresponding author due to ethical and project policy reasons.
